# Long non-coding RNA growth arrest specific 5 is downregulated in sepsis-ALI and inhibits apoptosis by up-regulating miR-146a

**DOI:** 10.1080/21655979.2021.2014619

**Published:** 2022-02-03

**Authors:** Jiaqiong Li, Hongyang Xu, Na Li, Wenjing Du, Junxiang Ti, Jingyu Chen

**Affiliations:** a Department of Critical Care Medicine, Xuzhou Central Hospital, Xuzhou Clinic School of Nanjing Medical University, Xuzhou City, Jiangsu Province, China; b Department of Lung Transplant Center, Wuxi People’s Hospital Affiliated to Nanjing Medical University, Wuxi City, Jiangsu Province, China

**Keywords:** GAS5, miR-146a, LPS, sepsis-induced acute lung injury, human bronchial epithelial cells

## Abstract

Long non-coding RNA (lncRNA) growth arrest specific 5 (GAS5) and microRNA (miR)-146a both have inhibitory effects on LPS-induced inflammation, suggesting the crosstalk between them. In this study, the expression of GAS5 and miR-146a in patients with sepsis-induced acute lung injury (sepsis-ALI), sepsis patients without obvious complications (sepsis) and healthy controls were studied by RT-qPCR. The role of GAS5 in the expression and methylation of miR-146a in human bronchial epithelial cells (HBEpCs) were studied by RT-qPCR and methylation-specific PCR (MSP), respectively. Cell apoptosis was analyzed by flow cytometry. We found that GAS5 and miR-146a were downregulated in sepsis-ALI and the expression of these two were correlated. LPS induced the downregulation of GAS5 and miR-146a in HBEpCs. In HBEpCs, overexpression of GAS5 increased the expression levels of miR-146a and reduced the methylation of miR-146a gene. Under lipopolysaccharide (LPS) treatment, overexpression of GAS5 and miR-146a decreased the apoptotic rate of HBEpCs. Moreover, the combined overexpression of GAS5 and miR-146a showed stronger effects. Therefore, GAS5 is downregulated in sepsis-ALI and inhibits cell apoptosis by up-regulating the expression of miR-146a.

## Background

Sepsis is a potentially life-threatening inflammatory immune response caused by bacterial, viral, fungal or protozoan infections [1]. Bacterial infection is the most common cause of sepsis [2]. Sepsis in severe cases may cause septic shock, which is related to high mortality rate [3]. In clinical practices, patients with sepsis usually develop organ failures, such as severe lung injury [4]. Without proper treatment, organ failures could result in deaths [5]. Even after active treatment, organ damages may still not be fully recovered, lead to reduced life quality [6].

The critical roles of certain molecular players in the pathogenesis of sepsis have been reported [7–9]. Functional analysis of these molecular factors may improve sepsis treatment. Long non-coding RNAs (lncRNAs) play crucial roles in sepsis by regulating the expression of disease-related genes [10,11]. Lipopolysaccharide (LPS)-induced inflammation plays pivotal role in sepsis [12]. LncRNA growth arrest specific 5 (GAS5) and microRNA (miR)-146a both have inhibitory effects on LPS-induced inflammation [13,14]. GAS5 upregulates KLF2 to suppress LPS-induced inflammation in ATDC5 chondrocytes [13]. MiR-146a inhibits the development of LPS-induced acute lung injury by suppressing the production and secretion of TNF-α, IL-6, and IL-1β [14]. Therefore, GAS5 and miR-146a may interact with each other to participate in sepsis. This study was carried out to study the crosstalk between GAS5 and miR-146a in sepsis-induced acute lung injury (sepsis-ALK).

## Methods

### Research subjects

This study enrolled 48 sepsis-ALI patients (28 males and 20 females; 54.5 ± 6.1 years old), 48 sepsis patients (20 females and 28 males; 54.6 ± 6.2 years old) and 48 healthy controls (20 males and 28 males; 43 to 67 years old, mean age 54.5 ± 6.1 years old) at Xuzhou Central Hospital between May 2017 and May 2019. The ethics approval was obtained from the Ethics Committee of this hospital (Ethics approval number: No. WPH1632). Inclusion criteria: 1) new cases; 2) patients were willing to participate. Exclusion criteria: 1) other clinical disorders were diagnosed; 2) patients with initiated therapy; 3) patients with blood relationship. Sepsis was caused by bacterial infections in all cases. Systemic physiological examinations were performed on all healthy controls to make sure they were in normal physiological conditions. All participants signed the informed consent.

### Plasma samples

After fasting for overnight (before therapy), blood (3 ml) was extracted from each participant and mixed with EDTA. Blood was centrifuged at 1,200 g at room temperature for 12 min to collect plasma.

### Human bronchial epithelial cells (HBEpCs)

HBEpCs (Sigma-Aldrich, USA) were cultivated in Airway Epithelial Cell Growth Medium (PromoCell). Cells were subcultured, and cells collected from passage 3–5 were used in the following experiments. HBEpCs were pretreated with 6 µg/ml LPS for 48 h to induce sepsis model. LPS treatment was performed by culturing cells in medium containing 0, 1, 3, or 6 µg/ml LPS for 48 h.

### Cell transfections

The full length cDNA of GAS5 (NCBI Accession: NR_002578), miR-146a mimic (5ʹ-UGAGAACUGAAUUCCAUGGGUU-3ʹ), as well as NC were synthesized by Sangon (Shanghai, China). GAS5 cDNA was inserted into pcDNA3.1 backbone vector (Invitrogen) to construct the expression vector of GAS5. HBEpCs were transfected with either miRNA and/or expression vector using Lipofectamine 2000 (Invitrogen). Briefly, miRNA and (co-transfection)/or vector was first mixed with Lipofectamine 2000 to prepare transfection mixture, which was used to incubate with the cells for 6 h. After that, fresh medium was used to wash cells for three times and cell culture was then performed in fresh medium for 48 h prior to the subsequent assays.

### RNA preparations

Direct-zol RNA Kit (ZYMO RESEARCH) was used to extract total RNAs from both HBEpCs and plasma samples. For LPS treatment, HBEpCs were treated with LPS at the dosage of 0, 1, 3 or 6 µg/ml for 48 h, followed by RNA extractions.

### RT-qPCR

Following the synthesis of cDNA samples using SSRT-IV system (Invitrogen) through the following thermal conditions: 25°C for 5 min, 55°C for 30 min and 85°C for 10 min, qPCRs were performed to detect the expression levels of GAS5 and mature miR-146a with 18S rRNA as the internal control. PCR procedures were: 95°C for 1 min, then 40 cycles of 95°C for 10 s and 58°C for 45 s. Primer sequences were: GAS5-F: 5ʹ-TTCTGGGCTCAAGTGATCCT-3ʹ, GAS5-R: 5ʹ-TTGTGCCATGAGACTCCATCA-3ʹ; 18S-F: 5ʹ-TAACCCGTTGAACCCCATT-3ʹ, 18S-R: 5ʹ-CCATCCAATCGGTAGTAGC-3ʹ; miR-146a-F: 5ʹ-TGAGAACTGAATTCCAU-3ʹ, miR-146a-F poly (T). The 2^−ΔΔCT^ method was used to process Ct values.

### Methylation-specific PCR (MSP)

HBEpCs were subjected to DNA extractions through conventional methods. Genomic DNA was converted and PCR was performed to assay gene methylation.

### Cell apoptosis analysis

HBEpCs collected at passage 3–5 were further incubated with 6 µg/ml LPS for 48 h. Cells were digested with 0.25% trypsin, followed by washing with cold PBS. After that, PI and FITC-annexin V staining was performed. Apoptotic cells were separated by performing flow cytometry.

### Statistical analysis

The preparation of images and the data analyses were performed using the software Graphpad Prism 6. Differences among groups were compared by ANOVA Tukey’s test. *P* (one-tailed) <0.05 was statistically significant.

## Results

### GAS5 and miR-146a were downregulated in sepsis-ALI

**Figure 1.**
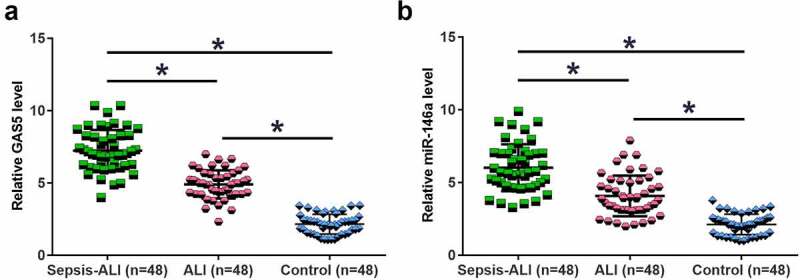
GAS5 and miR-146a were downregulated in sepsis-ALI. Expression of GAS5 (a) and miR-146a (b) in plasma samples from sepsis-ALI patients (n = 48), sepsis patients (n = 48) and healthy controls (n = 48) was determined by performing RT-qPCR. Each dot represents an average value of three technical replicates of qPCR. *, *p* < 0.05.

Altered gene expression may suggest function. To this end, the expression of GAS5 and miR-146a in plasma samples from sepsis-ALI patients (n = 48), sepsis patients (n = 48) and healthy controls (n = 48) were determined by performing RT-qPCR. The results showed that GAS5 (Figure 1(a)) and miR-146a (Figure 1(b)) were significantly downregulated in sepsis-ALI in comparison to that in the sepsis and control groups (*p* < 0.05). In addition, GAS5 and miR-146a were also downregulated in sepsis patients (*p* < 0.05). Therefore, GAS5 and miR-146a may participate in sepsis and sepsis-ALI.

### The expression levels of GAS5 and miR-146a were correlated across sepsis-ALI samples

**Figure 2.**
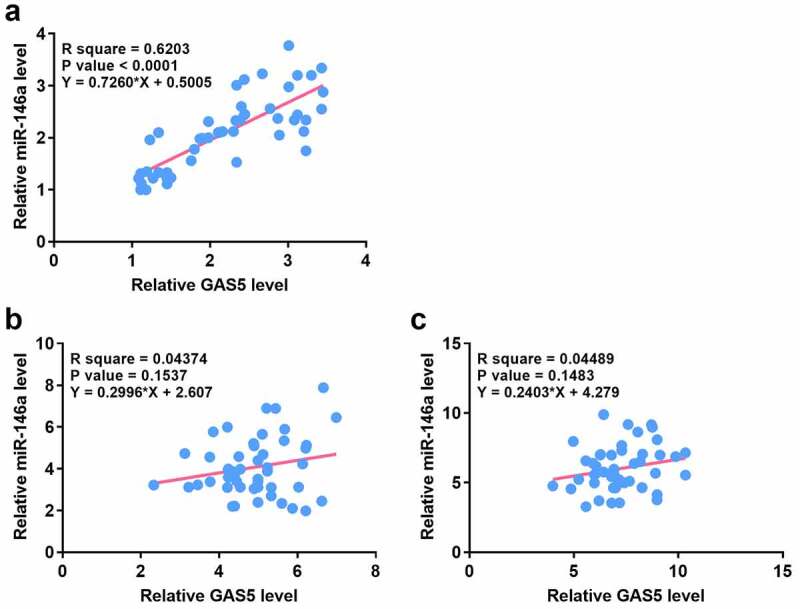
The expression levels of GAS5 and miR-146a were positively correlated across plasma samples from sepsis-ALI patients.

Correlations usually indicate interactions. Therefore, the correlation between GAS5 and miR-146a across sepsis-ALI (Figure 2(a)), sepsis (Figure 2(b)) and the control samples (Figure 2(c)) were analyzed. Correlation analysis showed that GAS5 and miR-146a were positively and significantly correlated across sepsis-ALI samples, but not sepsis samples and the control samples. These results indicated that GAS5 and miR-146a may have a crosstalk in sepsis-ALI.

Correlation analysis between GAS5 and miR-146a sepsis-ALI (a), sepsis patients (b) and control (c) plasma samples were analyzed by Pearson’ correlation coefficient. Each dot represents an average value of three technical replicates of qPCR.

### LPS treatment induced the downregulation of GAS5 and miR-146a in HBEpCs

**Figure 3.**
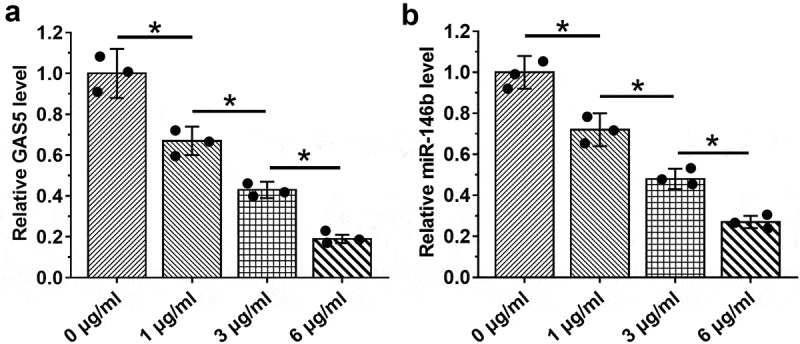
LPS treatment induced the downregulation of GAS5 and miR-146a in HBEpCs.

LPS contributes to sepsis and sepsis-ALI. Therefore, the effects of LPS on the expression of GAS5 and miR-146a were investigated. HBEpCs were treated with 0, 1, 3, or 6 µg/ml LPS for 48 h, followed by the measurement of the expression levels of GAS5 and miR-146a by performing RT-qPCR. It showed that LPS treatment decreased the expression levels of GAS5 (Figure 3(a)) and miR-146a (Figure 3(b)) (*p* < 0.05). These results indicated that altered expression of GAS5 and miR-146a in sepsis might be caused by LPS.

HBEpCs were treated with 0, 1, 3, or 6 µg/ml LPS for 48 h, followed by the measuring the expression levels of GAS5 (a) and miR-146a (b) by performing RT-qPCR. Mean± SD values of three biological replicates were used to present the data. Each dot represents one biological replicate. *, *p* < 0.05.

### Overexpression of GAS5 led to upregulated miR-146a in HBEpCs pretreated with 6 µg/ml LPS through methylation

**Figure 4.**
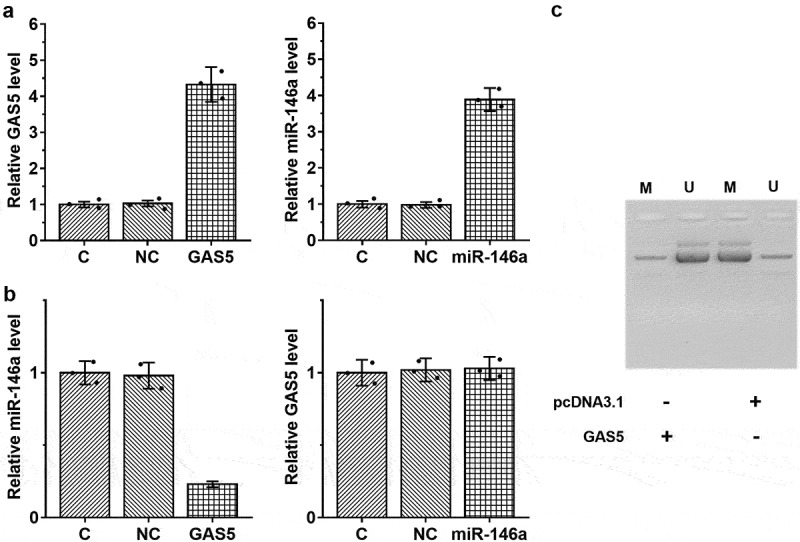
Overexpression of GAS5 led to upregulated miR-146a in HBEpCs through methylation. To analyze the interaction between GAS5 and miR-146a, HBEpCs were transfected with either GAS5 expression vector or miR-146a mimic. Overexpression of GAS5 and miR-146a was confirmed by performing RT-qPCR (a). The role of GAS5 and miR-146a in each others expression was studied with RT-qPCR (b). Involvement of GAS5 in miR-146a RNA gene methylation was studied with MSP. MSP products were subjected to agarose gel electrophoresis, followed by ethidium bromide staining to visualize the bands (c). Mean± SD values of three biological replicates were used to present the data. Each dot represents one biological replicate. *, *p* < 0.05.

The close correlation between GAS5 and miR-146a in sepsis-ALI indicates a potential interaction between them. To explore their interaction, HBEpCs were overexpressed with GAS5 and miR-146a (Figure 4(a), *p* < 0.05). Compared to the two controls, overexpression of GAS5 resulted in upregulated miR-146a, while miR-146a showed no role in regulating the expression of GAS5 (Figure 4(b), *p* < 0.05). Methylation analysis illustrated that overexpression of GAS5 increased the methylation of miR-146a gene (Figure 4(c)). Therefore, GAS5 may increase the expression levels of miR-146a through methylation.

### Overexpression of GAS5 and miR-146a decreased the apoptosis of HBEpCs

**Figure 5.**
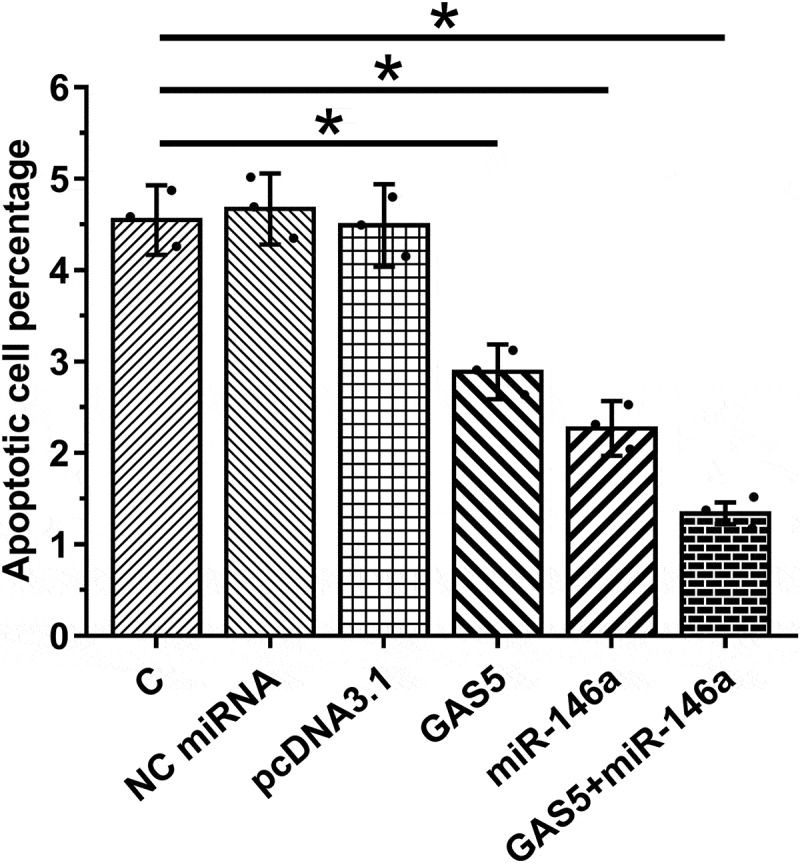
Overexpression of GAS5 and miR-146a suppressed the apoptosis of HBEpCs induced by LPS.

Cell apoptosis contributes to sepsis-ALI. Therefore, the role of GAS5 and miR-146a in cell apoptosis induced by LPS was investigated. Under LPS treatment, overexpression of GAS5 and miR-146a led to suppressed apoptosis of HBEpCs. Moreover, the combination of overexpression of GAS5 and miR-146a showed stronger effects (Figure 5, *p* < 0.05). Therefore, GAS5 may suppress cell apoptosis through miR-146a.

The role of GAS5 and miR-146a in regulating the apoptosis of HBEpCs induced by LPS was analyzed by cell apoptosis assay. Mean± SD values of three biological replicates were used to present the data. Each dot represents one biological replicate. *, *p* < 0.05.

## Discussion

The crosstalk between GAS5 and miR-146a in sepsis-ALK was investigated. Our data illustrated that GAS5 and miR-146a were both downregulated in sepsis-ALK. In addition, GAS5 may upregulate miR-146a through methylation to suppress the apoptosis of HBEpCs induced by LPS.

Previous studies have shown the involvement of GAS5 and miR-146a in LPS-induced inflammatory responses [13,14]. It was reported that overexpression of miR-146a suppresses the production and secretion of TNF-α, IL-6, and IL-1β to inhibit the development of LPS-induced acute lung injury [14]. In another study, Li *et al*. reported that overexpression of GAS5 upregulated KLF2 to reverse injuries in chondrocytes caused by LPS-mediated inflammation [13]. We also observed the decreased expression levels of GAS5 and miR-146a in HBEpCs induced by LPS treatment. In addition, overexpression of GAS5 and miR-146a inhibited the apoptosis of HBEpCs induced by LPS. Therefore, GAS5 and miR-146a may serve as potential targets to improve the conditions of sepsis-ALK patients.

Interestingly, we showed that GAS5 and miR-146a were downregulated in both sepsis patients without obvious complications and sepsis-ALK patients. Moreover, the decreases in the expression levels of GAS5 and miR-146a were higher in sepsis-ALK patients in comparison to that in sepsis patients. Therefore, sepsis may cause the downregulation of GAS5 and miR-146a. The continuous downregulation of GAS5 and miR-146a may in turn result in the development of lung injury in sepsis patients.

We showed that GAS5 could upregulate the expression of miR-146a in HBEpCs by increasing the methylation of miR-146a gene. However, we observed the significant correlation between GAS5 and miR-146a only across sepsis-ALK. Therefore, the reduction of GAS5 and miR-146a in lung tissues of sepsis-ALK patients may significantly affect their expression levels in plasma. In addition, the crosstalk between GAS5 and miR-146a may mediated by certain pathological factors.

This study only enrolled a small number of participants. Therefore, the conclusions should be verified by more studies with a larger size of participants. In addition, animal models are needed to further analyze the interaction between GAS5 and miR-146a *in vivo*. In recent years, an increasing number of studies have reported the roles of lncRNAs in ALK [15–18]. However, the function of lncRNAs in this disease remain unclear and more studies are needed.

## Conclusion

GAS5 and miR-146a were downregulated in sepsis-ALK and GAS5 may decrease miR-146a gene methylation to upregulate miR-146a, thereby decreasing the apoptosis of HBEpCs induced by LPS. We characterized a novel GAS5/miR-146a axis in sepsis-ALK. This axis may be targeted to treat sepsis-ALK. However, clinical trials are needed to test this possibility.

## Data Availability

The datasets used and/or analyzed during the current study are available from the corresponding author on reasonable request.

## Research highlights

1. GAS5 and miR-146a expression was altered in sepsis-ALI;
2. GAS5 upregulates miR-146a through methylation;
3. GAS5 suppresses cell apoptosis through miR-146a.
